# The importance of group factors in the delivery of group-based parenting programmes: a process evaluation of Mellow Babies

**DOI:** 10.3389/frcha.2024.1395365

**Published:** 2024-06-06

**Authors:** Jessica Tanner, Philip Wilson, Daniel Wight, Lucy Thompson

**Affiliations:** ^1^ Centre for Rural Health, Centre for Health Science, University of Aberdeen, Inverness, United Kingdom; ^2^ Centre for General Practice, Institute for Public Health Science, University of Copenhagen, Copenhagen, Denmark; ^3^ MRC/CSO Social and Public Health Sciences Unit, University of Glasgow, Glasgow, United Kingdom

**Keywords:** parenting programmes, group-based interventions, group processes, group context, maternal wellbeing, mother-infant relationship

## Abstract

**Introduction:**

The role of the group has been largely overlooked within evaluations of group-based parenting programmes. Group contextual factors, including size and level of homogeneity, may impact on essential group processes, such as group identification and cohesion, that are necessary to activate interpersonal change mechanisms and attain programme outcomes. This process evaluation of Mellow Babies, a 14-week attachment-based group parenting programme for mothers of infants aged under 18 months, explores how group context affected mother and practitioner experiences of the programme.

**Methods:**

In-depth interviews were conducted with fourteen mothers and three practitioners from three different Mellow Babies groups. Framework Analysis was employed to analyse data, using groups as cases within the framework matrix while preserving individual participants within each case. This allowed comparisons to be made within and between groups.

**Results:**

Four group contextual factors impacted on the quality of programme delivery: (1) group size; (2) level of group homogeneity; (3) pre-existing relationships; and (4) personalities within the group. These contextual factors affected the hypothesised intervention mechanisms: (1) fluid progression through the stages of group development; (2) a safe, non-judgemental, contained space; (3) social identification with group; (4) group cohesion; and (5) a culture of openness, support and empowerment.

**Discussion:**

Findings have implications for future delivery and implementation of group-based parenting programmes, for example, the importance of considering group composition during programme recruitment. Practitioners may also benefit from a stronger focus on group processes and enabling group dynamics within training, supervision, and the programme manual.

## 1 Introduction

The role of the group has been largely overlooked within evaluations of group-based parenting interventions (1, 2) despite qualitative research suggesting the centrality of group processes in supporting engagement and facilitating change [e.g., (3, 4)]. Alongside intervention content, interactions occurring within the group may be essential to the programme's mechanism of change (2), for example through normalising and validating challenges, and providing social support (2–6). Conversely, group processes can also diminish the effectiveness of a programme, decreasing parental engagement, undermining participants' wellbeing, evoking feelings of rejection and stigmatisation and reducing future help-seeking behaviours (4, 7, 8). As (9) pp. 28 states, “*…membership in a group promotes a range of positive social and psychological outcomes, but these benefits are not as positive as the effects of exclusion are negative*”. Group processes may therefore contribute to the 30% of families who do not show improvements after participating in parenting support programmes, even among programmes with an established evidence base (10). Participants who struggle to identify with the group, or experience challenges interacting with other members may not reap benefits from participating in group-based programmes (2, 11).

Elucidating the mechanisms of change and understanding how these mechanisms are influenced by context is an essential component of evaluating complex interventions and should be articulated within programme theory (12). Understanding how a programme may be affected by group context can offer valuable insight into the conditions under which the intervention is most effective (13). This not only helps optimise intervention efficacy (12, 14), but can also prevent what Grant and Hood (15) refer to as the “*crisis of replication*”, whereby an intervention may achieve desired outcomes when delivered in one group context but fail to produce effective results in another.

Borek et al. (2), proposed the Mechanisms of Action in Group-based Interventions (MAGI) framework, outlining two distinct pathways of change: the intrapersonal mechanisms of change, occurring on an individual level, and the interpersonal mechanisms of change, which are generated through interactions within the group. Contextual factors, including the wider implementation context, and participant, practitioner and group characteristics, have a dynamic and reciprocal influence on group processes, and subsequently the change mechanisms which are activated.

Another paper from this evaluation (under review) uses the MAGI framework to articulate a revised theory of change for the Mellow Babies group-based parenting programme, specifying the key interpersonal change mechanisms, namely: (1) normalisation through social comparisons; (2) cognitive reframing through group feedback; (3) peer support; and (4) social and experiential learning. Findings from interviews with practitioners and participating mothers indicated that interpersonal change mechanisms mediated intrapersonal change mechanisms, which in turn mediated programme outcomes.

Alongside mediational models within a theory of change, consideration of any moderated mediations is also needed (16). Moderated mediation occurs when the mediator pathway is contingent on the presence or absence of a moderator variable. In this way, group context may represent a form of moderated mediation: some interpersonal change mechanisms may be dependent on specific group characteristics or group processes, including group size, level of group homogeneity and group cohesion (8, 17–19).

In addition to group-level characteristics, including size, level of homogeneity and group cohesion, identification with the group at an individual level may also be a prerequisite for change. Social identification, derived from Tajfel and Turner's (20) social identity theory, is defined as “*positive emotional valuation of the relationship between self and ingroup*” (21) pp. 599. (The ingroup refers to the social group that an individual perceives they belong to. This can be related to gender, culture, religion, or other social category, such as “mother”). Individuals experience social identification with a group if they feel solidarity with other group members, perceive group membership to be significant to their self-concept, and have a positive perception of group membership (22). Social identification with the group is greater when participants feel a sense of shared experiences with other members (23). Higher levels of social identification with the group are associated with increased retention and improved programme outcomes (23–26). Social identification may be particularly important during significant life transitions (24), including facilitating adjustment to motherhood (27), where women often experience a loss of identity and prioritise their baby's needs over their own (28). This can be exacerbated by postnatal services predominantly focused on infant, rather than maternal, wellbeing (29). Equally, a lack of identification with the group can result in being “othered”, which can occur via the formation of “in-groups” and “out-groups” within group interactions, or through “self-othering”, via the participant's own perception (7, 8, 30, 31). Understanding more about what facilitates and impedes group identification processes may help reduce adverse impacts of group-based programmes (32).

This research aimed to explore how group context impacted on mothers' and practitioners' experiences of Mellow Babies, including on their engagement with the programme, their perceptions of group cohesion and identification, and their perspectives on the “effectiveness” of the group. Although key intended outcomes, specified in the theory of change, were improved maternal wellbeing and parent-infant attachment, the “effectiveness” of the group was based on participants' subjective perceptions, following the definition of “successful” group experiences outlined by Rogers [(33); pp. 340]:

“*If it was a meaningless, dissatisfying experience, or a hurtful one from which they are still recovering, then for them, this was certainly not a successful group. If, on the other hand, most or all members still feel that it was a rewarding experience which somehow moved them forward in their own growth, then for me it deserves the label of a successful group.*”

Research suggests that groups who experience greater satisfaction with relationships and cohesion are significantly associated with programme outcomes (34).

### 1.1 Intervention

Mellow Babies is a manualised, attachment-based parenting programme which aims to improve parental wellbeing and foster healthy parent-infant relationships. It is usually delivered as a targeted intervention to mothers who have psychosocial difficulties which may impact on the development of their infant (aged up to 18 months). The group consists of fourteen weekly sessions which last approximately five hours, with a shared lunch. Mothers attend the group with their babies who are looked after by childcare workers while they participate in sessions which include structured activities and reflective group discussions. Strength-based video feedback is used to develop maternal sensitivity and attunement, and mothers are provided with activities (e.g., baby massage) to try at home with their infant. The programme is delivered by two or three trained practitioners who receive regular supervision over the course of the group.

**Figure 1:**
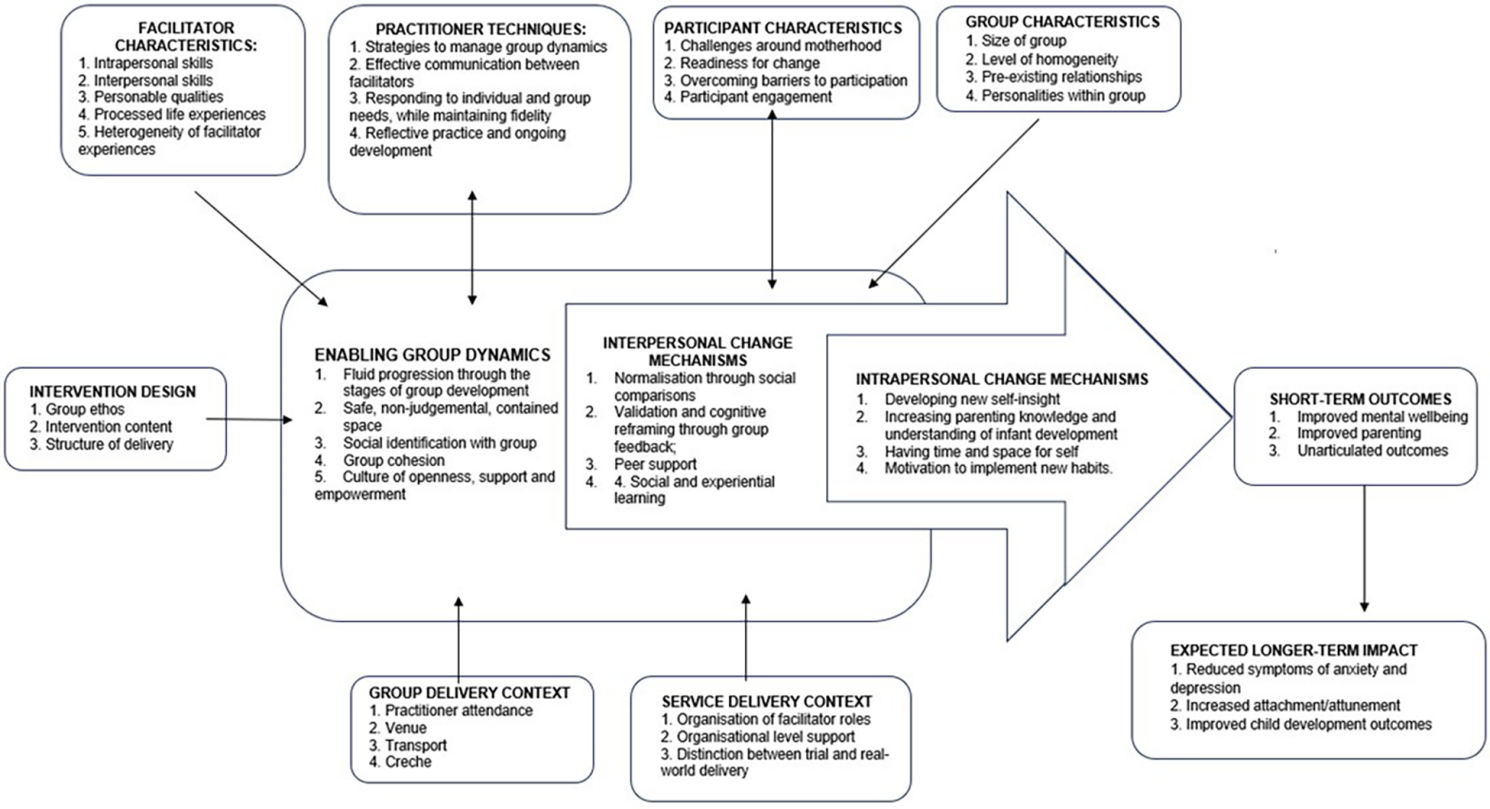
Theory of change for Mellow Babies.

The research presented in this paper derives from a process evaluation conducted alongside a Randomised Control Trial (RCT) conducted in the Highland Council region of Scotland (IRAS ID: 157028). Mothers were referred to the trial via health professionals, including health visitors and GPs, or self-referred by responding to letters or adverts on social media or in the local community. Mothers were screened using the Hospital Depression and Anxiety Scale (HADS) and needed to score above the 85th centile for anxiety and/or depression within the UK female population (35). Eligible mothers were randomised to either Mellow Babies or Care as Usual (CAU) on a 1:1 allocation. A theory of change is depicted in Figure 1 below.

## 2 Method

### 2.1 Participants

Post-group interviews were conducted with fourteen mothers and three group practitioners who participated in three Mellow Babies groups delivered between March and December 2022. All mothers who attended at least one Mellow Babies session were invited to participate in an interview. 3 mothers opted not to participate, two who withdrew and one who completed the programme. Characteristics of mothers, practitioners and the group context are shown in Tables 1–3 below.

**Table 1:** Characteristics of participating mothers.

Participant ID	Group	Age bracket	Number of children	Interview conducted
M1	1	31–35	1	In-person
M2	1	26–30	2	In-person
M3	1	41–45	4	In-person
M4	1	31–35	2	Video call
M5	1	36–40	1	In-person
M6	2	31–35	2	In-person
M7	2	21–25	3	Telephone
M8	3	31–35	1	In-person
M9	3	31–35	1	In-person
M10	3	31–35	1	Telephone
M11	3	25–30	1	In-person
M12	3	25–30	2	In-person
M13	3	21–25	1	Telephone
M14	3	25–30	1	Telephone

**Table 2:** Characteristics of participating practitioners.

Participant ID	Groups delivered	Age	Mother themselves	Experience delivering groups	Experience working with mothers
P1	1, 2, 3	30–40	Yes	Yes	Yes
P2	1, 2, 3 (left midway through delivery of group 3)	20–30	No	No	Yes
P3	3	Over 50	Yes	Yes	No

**Table 3:** Characteristics of each group.

	Group 1	Group 2	Group 3
Location	City	Small Town	City
Delivery day	Weekday	Weekend	Weekday
Number attending at least 1 session	6	4	8
Number remaining enrolled in intervention	5 (83%)	3 (75%)	7 (88%)
Number of sessions delivered	14 out of 14	10 out of 14	11 out of 14
Number of Group Practitioners	3 1 left (unplanned) midway	2	3 1 left (planned) midway
Age	Range 28–44	Range 23–32	Range 21–32
Relationship Status	4 in relationships 1 separated from partner during group	All 3 in relationships	5 in relationships 1 single parent 1 separated from partner during group
Ethnicity	All White British	All White British	4 White British 1 Asian British 1 Spanish 1 White American
Previous connections	2 cases of practitioner/mother acquaintance	2 mothers known to each other Practitioner knew 1 mother in professional capacity	No real connections, 1 “recognised face from school”

#### 2.2.1 Data collection

Individual, semi-structured interviews were used to explore mothers' and group practitioners' experiences of group processes. Mothers were interviewed after they completed the programme, and group practitioners were interviewed both at the end and at the midway point of delivery in order to capture perspectives on the earlier stages of group development that may have been forgotten by the end of the 14-week intervention. Interviews were conducted in-person, via telephone or via videoconferencing software, depending on the participants' preference.

An interview schedule was used, following the structure outline by DeJonckheere & Vaughn (36), whereby each topic area began with a “grand tour” question, followed by a set of more specific “core” questions. Additional follow-up questions and discussions were then based on participants' responses. This flexibility for deeper exploration was essential given the diversity of individual experiences. Interviews were recorded and transcribed verbatim prior to analysis. Ethical approval for this study was obtained from the East Midlands—Nottingham 1 Research Ethics Committee (Ref: 18/EM/0304).

#### 2.2.2 Data analysis

Data were analysed inductively using Framework Analysis (37), providing a systematic and transparent method commonly used with semi-structured interviews (38, 39). Analysis followed the seven steps outlined by Gale et al. (39): First, verbatim transcripts were produced for each of the interviews. Second, familiarisation with the data was achieved by listening to the audio recordings and reading the transcripts. Third, preliminary codes were identified across all transcripts. Fourth, the development and refinement of an overarching thematic framework was used to construct the framework matrix. These initial themes were based on broad descriptive rather than analytic themes (38) to permit comparisons between cases on the matrix. Fifth, indexing, allowed identification of corresponding themes within the data using N-Vivo software (version 12) Sixth, charting allowed the data to be placed onto a framework matrix using N-Vivo. The “cases” on the framework matrix were the three groups (39), with individual participant data preserved within each case allowing comparisons to be made both within and between groups. Finally, analytic sub-themes were identified based on mapping and interpretation of the framework matrix.

## 3 Results

A revised theory of change for Mellow Babies, incorporating group contextual factors, is depicted in Paper 1 (submitted concurrently). Findings will be presented based on two components of the programme theory of change (Figure 1, above): (1) Group Contextual Factors and (2) Enabling Group Dynamics. These were compared across all three groups.

**Figure 2:**
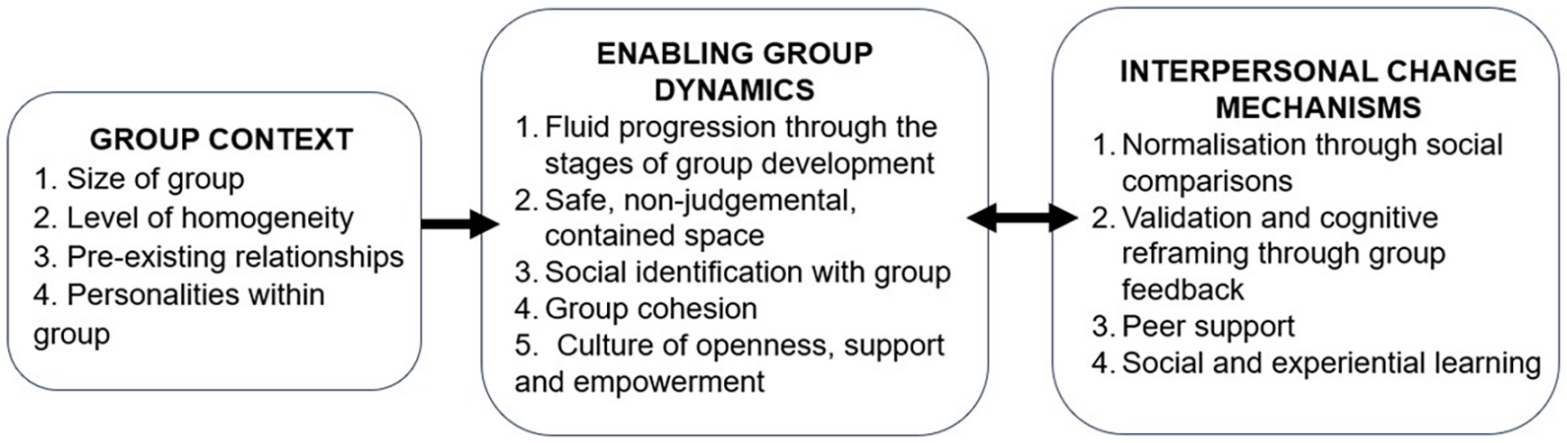
The relationship between group context, enabling group dynamics and interpersonal mechanisms of change.

There were notable difference in the perceived effectiveness of the three groups, with mothers in Groups 1 and 3 unanimously citing benefits from participation. In contrast, one of the three Group 2 mothers interviewed, stated that she experienced minimal benefits from the group, while the other two perceived that the programme had no positive impacts on their life, “*I didn't get anything from it, no*”. (M6; Group 2) and cited adverse effects. While individual factors, such as readiness for change, may have played a role, comparisons of mother and practitioner perceptions between the three groups demonstrated that Group 2 was perceived to be less successful owing to a range of group contextual factors which inhibited enabling group dynamics (Figure 2).

### 3.1 Group contextual factors affecting the effectiveness of mellow babies

#### 3.1.1 Group size

Group size was perceived to have a significant impact on the effectiveness of Mellow Babies, affecting participant engagement, programme fidelity, group cohesion and group dynamics. The group of three (following the withdrawal of one participant) in Group 2 was not perceived as a viable group by either mothers or practitioners:

“*Three mums is too little, and even four was too little of a group.*” (P1; Group 2).

“*It actually didn*’*t feel like a group.*” (M6; Group 2).

In contrast, mothers and practitioners across Groups 1 and 3 were happy with their group size of five and seven mothers, respectively. Group 1 mothers perceived that their group of five was the “*perfect number*” (P1; Group 1): there were enough members to prevent anyone feeling “*under forensics*” (M3; Group 1), yet all members were able to share their experiences with sufficient depth to support group cohesion, and enable the interpersonal change mechanisms of normalisation, validation, and cognitive reframing (concurrent submission). Although most mothers recommended a group size of between five and ten members to support participation, there may be individual preferences. Practitioners reported that one mother in Group 2, who did not participate in an interview, preferred the smaller group: “*She said she quite liked it because there was less people, she felt more able to speak*” (P1; Group 2). They also described how one of the quieter members of Group 3 “*really came out of herself*” (P3; Group 3) during a week where only four mothers were able to attend. Akin to Yalom and Leszcz's [(40); pp. 292] assertion that members of small groups may participate due to “*a sense of obligation rather than true alliance*”, mothers in Group 2 reported feeling pressure both to participate in group discussions and to maintain attendance, as they were aware the session would get cancelled if they were absent, and the group would be terminated if they withdrew from the programme. This led to feelings of resentment among mothers, and caused tensions within the group when other mothers were absent and sessions were cancelled.

The small size of Group 2 also impacted on programme fidelity. As group discussions were often stilted, practitioners described having to “*fluff the sessions up*” (P1; Group 2). They also described how they would “*would do more chatting to try and fill the time up*” (P1; Group 2), but ultimately felt this detracted focus from mothers' experiences and “*dilutes*” programme content. A further issue with Group 2 was that challenges with interpersonal dynamics were amplified within a small group, when the same challenges would have been diluted with more members. Furthermore, with three members, group discussions could end up feeling like two vs. one when sharing opinions, and on occasions, this majority stance could inadvertently reinforce maladaptive views.

#### 3.1.2 Level of group homogeneity

Each group comprised a variety of class and educational backgrounds, and Group 3 also contained a mix of culture and religions. Having this diversity of experiences helped mothers accept the universality of life's challenges:

“*I was just worried everyone is going to be the same and I’m going to be the one left out…But you realise as well that even though they have such different experiences, it doesn't necessarily mean that their journey is easier than yours or harder than yours.*” (M10; Group 3).

Mothers and practitioners felt that diversity within the group supported effective delivery, providing a variety of perspectives during group discussions, and forming connections at a more meaningful level:

“*I think because of the experiences we’ve all got, we’ve bonded on quite a deep level, whereas [with] similarities we may have just bonded on a surface.*” (M4; Group 1).

“*I don*’*t know if it*’*s just the diversity that actually works. Sometimes if you’ve got people who are too similar and they’ve all grown up in the same area and have got nothing that*’*s different other than they have different dads for their children, it probably makes it a bit too insular. But the fact that people have such varied stories.*” (P3; Group 3).

It was also perceived to be helpful to have a mix of primiparous and more experienced mothers: new mothers could benefit from gaining advice, and dispensing this advice increased experienced mothers’ feelings of competence.

While some heterogeneity was beneficial, the general consensus of mothers and practitioners was that with heterogenous groups there needed to be a “*really good mix*” rather than a majority and “*small minority*” (P1; Group 1), which could lead to “othering”. There also needed to be some degree of homogeneity in order for mothers to identify with other group members and relate to their experiences.

The critical group characteristics where homogeneity is necessary may depend on the participant and the group. Within these three Mellow Babies Groups, single parent status or having a baby with developmental difficulties were not perceived to be important by the mothers involved. In both circumstances, although it initially evoked difficult feelings, being part of a diverse group helped mothers gain acceptance of their own situations. For example, one single mother described her realisation that having a partner may not make raising a baby easier:

“*I was the only single mum and they were all talking about their husbands but then it was actually coming to light more and more each week that actually their partners weren*’*t helping…Realistically they were in the same boat as me.*” (M11; Group 3).

Similarly, practitioners found the inclusion of a mother of a child with undiagnosed developmental difficulties “*really tricky*” (Practitioner 1; Group 3) and were concerned about her wellbeing during infant development discussions. However, the mother herself found that being around typically developing infants and being involved in discussions on infant development forced her confront the reality of the situation. She was able to address and process her feelings, and reached a state of acceptance that her infant was different.

“*I think because of the fact that all the other kids were developmentally normal, I think that helped me a little bit as well.*” (M12; Group 3).

Heterogeneous participant characteristics which impeded group identification and cohesion within the three groups studied included participants' age and the level of life adversity they were experiencing. While mothers generally felt that having sufficient life experience and being at a similar stage of life was more important than age *per se*, one mother in Group 3 reported feeling slightly isolated from the rest of the group due to her younger age, which made her a minority:

“*I think because I’m a lot younger, I didn't really get as close to them all as everyone else.*” (M13; Group 3).

Practitioners also perceived that group members needed to have experienced a comparable level of adversity within their life. The high level of cohesion within Group 1 was attributed in part to them being: “*all on a level, there wasn’t anybody that had a really chaotic home life, for example, where it was just off the scale. I think they all fit quite well together*”. (P1; Group 1). In contrast, within Group 2, one mother perceived her difficulties to be less than the other mothers in the group which made her question her fit in the group:

“*I don*’*t have a past like the other two women that were there, so I was shocked when I heard their life stories, I thought, “Oh my goodness. Am I meant to be here then?” I was thinking I* “*Oh god, is this meant to be for me?*” *Maybe that*’*s why I’m not getting anything out of this.*” (M6; Group 2)

#### 3.1.3 Existing relationships

Prior to starting the programme, a common apprehension was knowing someone else in the group:

“*[T]hat was one thing that I didn't really want, I didn't want to know anyone, that I was going to go and then maybe hold back, sort of thing.*” [M4; Group 1].

These anxieties, shared by practitioners, were exacerbated for Group 2 which was recruited from a small town.

Within Groups 1 and 3, delivered in a city, existing relationships between group members were minimal and were at an “acquaintance” level, so had minimal impact on group dynamics. However, Group 2 was affected by two sets of prior relationships: two mothers who were at school together and shared the same social network, and between a practitioner and a mother, who had previously worked together. These existing relationships inhibited participants from making personal disclosures:

“*I wouldn*’*t go into a group again knowing someone… I didn*’*t like that actually because I don*’*t think I shared sometimes as much.*” (P1; Group 2)

“*I think they maybe held back on a few things…they would open up more when the other one wasn*’*t there.*” (P2; Group 2)

Practitioners described how these two mothers could often interject within each other's stories, particularly during the Life Stories session, and direct interactions to each other, rather than the whole group. This was likely amplified by the smaller group size:

*When they were talking about their life stories, and they were like, “oh you remember so and so” or “do you remember…?”* (P2; Group 2)

The prior working relationship of the mother and practitioner also affected the practitioner-mother dynamic, requiring the practitioner to redefine professional boundaries. The mother had more professional experience than the practitioner and altering the professional-service user relationship appeared challenging, with the mother questioning the practitioner's skillset and practice.

A final impact of existing relationships was that participants lost agency over what was shared within the group, as within both sets of existing relationships examples were given of disclosures made about the other party. As the practitioner described, she found this difficult because “*it wasn't on my terms*” (P1; Group 2).

#### 3.1.4 Personalities within group

Personal qualities of the mothers, including their openness, self-awareness and empathy, impacted on the group dynamics. If one or more mothers made intimate disclosures, this set a precedent for the future culture of the group and encouraged wider sharing. While quieter members may have found it more difficult to participate, if they were in a group where other mothers were self-aware and empathic the group would actively encourage their participation. One practitioner described an introverted mother within Group 3 who may have withdrawn from the programme had the group not been so inclusive and accepting:

“*If there was anybody that was a little bit harder personality or a little bit more abrupt, I think maybe she would have felt more out of place possibly. I think because they are just nice people, they all care, and you could see they were making the effort to try and include her.*” (P3; Group 3)

In contrast, the presence of a louder participant who had little self-awareness or awareness of others could dominate the group and lead to a quieter member having “*her voice taken away a few times*” (P1; Group 2). This was evident from both practitioner and mother perspectives, and may have been exacerbated by the smaller group size and the relative inexperience of the practitioners:

“*You couldn*’*t speak, one of them, you couldn*’*t speak because…she*’*s very opinionated and she’ll say it herself that she just speaks and speaks, and you never really got a word in with her. She overtook everybody, she just had that personality.*” (M7; Group 2)

### 3.2 The impact of group context on enabling group dynamics

#### 3.2.1 Fluid progression through the stages of group development

Effective groups progressed smoothly through the five stages of development outlined by Tuckman (41): forming, storming, norming, performing and adjourning. However, participant engagement could impact on this progression, when sporadic attendance and participant dropouts (including practitioners) could inhibit the “forming” stage of group development. This was particularly the case for Group 2, where there were lots of interruptions during initial weeks causing the group to lose momentum.

“*We did one week, then we were off a week, then we were in a week, then there was the two-week break, then it was cancelled because nobody could come.*” (P2; Group 2)

As the group started to form, practitioners described using techniques to unmask defences, and it was only when all participants felt able to share openly that the group started to fully cohere and enter into the performing phase:

“*We did have two that were more quiet and shy, so it just took them a little bit longer. The quicker we picked up on that, the more we could focus on them a little bit, to keep pulling them in. I think when they started talking, that*’*s where it all gelled together*” (P1; Group 1)

Informal conversations through a WhatsApp group chat (an instant messaging service) helped deepen group bonds, and a sense of group identity developed through inside jokes. This was perceived to be particularly helpful during the group forming stages, providing opportunities for quieter members to “*come out a little bit more*” (M12; Mother Interview), developing their confidence to participate.

“*I think the fact that we had a group chat as well gave them a bit more opportunity to say their piece.*” (M12; Mother Interview)

The WhatsApp group was utilised differently by each group. Group 2, the least cohesive, used it purely to discuss practical matters, for example, whether they were able to attend sessions. For Group 3, a cohesive group, it provided a platform for general support and friendship, with mothers regularly checking in and sharing advice between sessions. However, the WhatsApp group was used more fully, and therefore likely to be most effective, in Group 1, where mothers used the group to continue reflecting on session content with each other. Mothers found this particularly helpful as they developed new insights from continued reflection outside of the group:

“*I probably do come across as quite quiet…I think it*’*s because you’re trying to process it. But I’m not very good until I'm kind of like at home and I've got time…it*’*s so good because like in the WhatsApp group, like we can talk about stuff like that* [new reflections]*, which is really nice.*” (M3; Group 1)

Within these groups, practitioners were not part of the WhatsApp group. In other service delivery contexts, practitioners are often involved in the group chat and may set up the WhatsApp group themselves.

By the end of the programme, Group 1, identified by practitioners as the most cohesive group, required little input, with mothers themselves adopting the role of practitioners and facilitating discussions:

“*We literally said hardly anything in that last session. They all spoke for themselves. They asked each other questions about things they were talking about…It*’*s amazing when you see that happen.*” (P1; Group 1)

However, the downside of this was that mothers in Group 1 really struggled with the adjourning stage of the group, feeling “*bereft*” and entering into a “*downward spiral*” without the “*safety net*” of the group (M3; Group 1).

#### 3.2.2 Safe, non-judgemental and contained space

In order to actively participate in shared reflection, mothers needed to perceive the group to be a safe space where they were able to acknowledge difficult feelings and struggles, yet still feel accepted by the group. The group agreement was felt to be pivotal in creating a sense of safety by communicating clear expectations about the boundaries within and outside the group. Understanding that they had self-agency over their participation and were able to opt out of sharing when they did not feel comfortable helped this sense of safety.

A larger group size supported the creation of a non-judgemental space, increasing the likelihood of heterogeneous opinions and acceptance of different viewpoints. Sharing vulnerabilities, particularly through the Life Stories session (where participants recounted their life stories) was a great leveller for mothers from different backgrounds, helping them develop new empathy and understanding for each other, and overriding any preconceptions they may have held about mothers from a different culture, class, background etc.

“*I wouldn*’*t ever associate myself with them… I don*’*t know how to say this without sounding like a snob. It was just the way that they live, they swear, their language… now I understand them better so it made me realise that I was judging when I shouldn*’*t have been…Like it*’*s the same, we’re the same.*” (M6; Group 2)

The only contentious topic where mothers were not able to be accepting of other perspectives was within Group 2, where cultural differences over the roles of women caused friction. In this case, practitioners themselves described challenges distinguishing between cultural beliefs and abusive behaviour:

“*We had somebody that was very, very against toxic masculinity, but then we have somebody who is married to someone whose belief is that women shouldn*’*t work, women have to do this, women have to do that. I think a lot of the conversation was abruptly stopped because it just felt like it was just conflicting opinions it was almost hard to balance it.*” (P1; Group 2)

This may have been exacerbated by the smaller group size which amplified the impact of any mothers who could be forthright in expressing their views and offering judgement on others' situations. Practitioners reported that one mother made prescriptive comments such as “*you shouldn't allow yourself to…*” (P2; Group 2) which restricted further sharing from participants:

“*There*’*s one mum that could be quite blunt with the others at times…we’ve kind of tried to bring that back so it doesn*’*t, not coming across like you’re looking down on them, you disagree with them in that way.*” (P1; Group 2)

Pre-existing relationships could also thwart the creation of a non-judgemental space, as group members who knew each other prior to the group, or those who shared mutual connections, were not able to be objective in how they perceived each other:

“*I don*’*t feel like I’ve got an objective view on them because I already know them.*” (P1; Group 2)

Similarly, sharing, validating and advising was difficult when group members had their own perspectives on the experiences being shared, for example, where a participant's former abusive partner was friends with another mother. Participants felt safest within the group environment when it was a contained space, with no connection to their world outside the group:

“*They’re a safe place to share because they’re not a friend that knows another friend that you’re gurning about.*” (M4; Group 1)

#### 3.2.3 Social identification with the group

Several group contextual factors impacted on social identification with the group. First, group size, as a “*critical mass*” (Yalom; 1975) was required for mothers to feel social identification with the group. There needed to be enough members so mothers would have a “*chance of somebody being slightly similar*”. (P2; Group 2), but sufficiently few members that mothers were able to fully share their experiences during discussions in order to foster connections with others.

Second, there needed to be sufficient homogeneity so mothers were able to feel a sense of identification with the group. As discussed earlier, age, culture, class, education, and level of life adversity were key characteristics which could impede group identification if mothers felt different to the group. Participants were more likely to withdraw from the programme under these circumstances, with two out of the three mothers who withdrew being perceived as “different” from the rest of the group due to class and educational differences:

“*We had six mums at one point and one left. The one that left, I don*’*t think she fit into the group quite so easily, I have to say. She was very different… I think she was at a different point in her life, professionally and… I also want to say maybe financially.*” (P1; Group 1).

Practitioners also perceived that group identification was higher in groups where members had the same motivation for change, where they all “*want to be there*”, and “*genuinely want help*” (P3; Group 3). Most mothers enrolled in Mellow Babies because they wanted to improve their mental health, relationship with their baby, or build their social support network. Some mothers described intense struggles with low mood or anxiety, which were significantly affecting their life and their relationship with their infant:

“*I was really defeated at the time. Like I was just, as I say just the lowest, I think I’ve ever been. And I just needed, just was hoping to be able to, pick myself up again… I just knew that I needed help and it just seemed like a lifeline at the time.*” [M11, Group 3]

Mothers wanted to connect with other mothers who were experiencing similar struggles, particularly as they often did not identify with mothers attending community mother-baby groups:

“*You go to other groups, and you think everybody is just very happy and content and everything. When this letter came to me, I was like, if I’m going there, I'm going to see mums that are feeling anxious too, or overwhelmed.*” [M8, Group 3]

Mothers were able to relate to each other through similar feelings and difficulties, and found comfort in being able to share their experiences of low mood, anxiety, poor body image, loneliness and fatigue with people who could understand and validate their experiences:

“*You can make connections with people knowing what they’ve been through, and you can make connections with your own life.*” (M5; Group 1)

#### 3.2.4 Group cohesion

Group cohesion was formed through fostering individual connections, alongside relational closeness as a group through group identification. A smaller group size was more manageable for forging close individual relationships. While Group 3 members (the group of 7) all reported identifying with the group, Group 1 (the group of 5) appeared to feel a much stronger group cohesion, describing themselves as “*five little best friends*” (M5; Group 1):

“*We got to know each other individually. Although we do really well* [as a group], *it*’*s nice to spend time with each other individually.*” (M1; Group 1).

Although individual relationships could form within larger groups, these have the tendency to feel like cliques or sub-groups (18). While Group 3 was not experienced as “cliquey” by any of the mothers and remained a cohesive group, it was clear that mothers and practitioners perceived the presence of two sub-groups, “loud” and “quiet” mothers.

#### 3.2.5 Culture of openness, support and empowerment

The final characteristic of enabling group dynamics was a culture of openness and empowerment. Sharing of personal experience by practitioners was perceived to play a pivotal role in creating a culture of vulnerability and acceptance:

“[It] *allowed us to open up to them more because we knew that they’d been through stuff as well and they weren't perfect, because we weren't perfect.*” (M6; Group 1)

Similarly, the personalities and level of openness of group members also contributed to this culture. The “*gentle*” nature of the programme was also appreciated: instead of being told what to do, mothers were encouraged to generate their own advice and ideas and were empowered in their ability to “*help each other*”. (M17; Mother Interview). Giving and receiving support facilitated individual and group cohesion.

For some mothers who had unprocessed trauma, who were currently experiencing significant life stress, or who still felt raw emotions from topics being discussed, it was more difficult to share during group discussions. When group members were able to be open with the group about why they were finding it difficult to participate, the group was able to be accepting and supportive. However, when defences such as anger or deflection were used, this was more difficult for the group to tolerate and could restrict wider discussions. A mother in Group 2 described “*walking on eggshells*” (M7; Group 2) around a reluctant group member, feeling like the group only began to enter the “performing” phase after this member had withdrawn from the group. Mellow Babies practitioners also perceived that group coherence was higher in groups where members were all “*at a certain point in their journey*” (P1; Group 2) where they were able to discuss past adversity.

Mothers' level of openness had a direct and reciprocal impact on cohesion, as mothers bonded through sharing their vulnerabilities which “*broke down a lot of barriers*” and put “*everybody on a level playing field*”. (P1; Group 1). Mellow Babies was often compared to other community baby groups, which were perceived as impersonal as conversations stayed on a superficial level. In contrast, the deep sharing in Mellow Babies helped mothers to feel connected at both an individual and group level.

## 4 Discussion

Understanding context-mechanisms-output configurations is an essential part of realist evaluation (13, 42). Previous research has suggested that group-based parenting programmes are not universally beneficial (10). While this may in part be due to individual readiness for change (43–45), findings from this process evaluation demonstrate how group context can impact on the interpersonal change mechanisms within Mellow Babies, affecting programme outcomes.

With the three groups studied, four group contextual factors impacted on the quality of Mellow Babies delivery, via the enabling group dynamics and interpersonal mechanisms of change. First a “*critical mass*” of at least five participants is needed, with the optimal size of reflective groups being between five and ten members (40, 46). The initial group size also needs to be able to accommodate participant dropout and low attendance without dipping below the optimal size. Yalom (40) pp. 293 states that “*Group size is inversely proportional to interaction*”: smaller groups may require effortful participation to maintain discussion flow, whereas larger groups may compromise and limit member contributions. Corresponding with findings from Berry et al. (47), a smaller group could feel more exposing, amplify challenges with group dynamics and compromise programme fidelity (47). While not explored within this process evaluation, programme fidelity can also be compromised when groups are larger than the recommended size (48).

Second, the level of group homogeneity can have a significant impact on group processes and change mechanisms. While homogenous groups: “*gel more quickly, become more cohesive, offer more immediate support to group members, have less conflict, and provide more rapid relief of symptoms*” [(40); pp. 272], group discussions may remain “*superficial*”, and they are therefore a “*less effective medium*” for inducing therapeutic change. Lonergan (49) pp. 114 advocates the importance of “*balance between homogeneity and heterogeneity*”. Heterogeneity can provide a diversity of perspectives and experiences, generating greater discussion and prompting deeper reflection. However, some degree of homogeneity is necessary to foster connections between mothers and ensure they feel some identification with the group. According to the mothers interviewed single parent status or having a baby with developmental difficulties were not perceived to be important characteristics for homogeneity. However, heterogeneity in age or experiences of adversity inhibited mothers from identifying with each other. Feeling their experiences are dissimilar to others' can reduce participants' sharing, partly to self-protect against being marked as “different” but also to protect the group, particularly if they are worried about group members being able to tolerate their traumatic experiences (8, 11). Indeed, several mothers within this study reported sadness and distress at hearing some of the other mothers' adversity, aligning with previous research that parents may be “triggered” by other parents' stories, and disclosures may cause parents to distance themselves from the group as a whole, or from certain group members (8, 44, 50). Although none of the mothers interviewed within this study disclosed feeling a greater level of difficulties than the rest of the group, previous research has indicated that when parents perceive their difficulties to be greater than the rest of the group it can increase stress and reduce self-esteem and wellbeing (31, 51).

Thirdly, pre-existing relationships could inhibit participants from sharing their experiences. Depending on the nature of the prior relationships, this had the potential to cause tension within the group, or be experienced as cliquey by other mothers. Finally, personalities within the group impacted on the effectiveness of Mellow Babies, and mothers with low self-awareness or self-control could dominate the group and intimidate quieter members from participating. It is worth noting that practitioners in this study were new to delivery of Mellow Babies, and therefore experienced practitioners may have been more adept at managing some of these dynamics. Hargaden [(52); pp. 285] argues that while empathy is essential for therapeutic change, and group members should be “*carefully considerate of one another*”, the “overuse” of empathy could restrict exploration of more primitive feelings and inhibit the group from making the compassionate challenges to support cognitive reframing.

These group context factors impacted on the enabling group dynamics and development of each group. First, corresponding with the findings of Buston et al. (8), low engagement with the group, through sporadic attendance and discussion participation, could prevent the group from entering into the “performing” phase of group development which has the greatest potential for therapeutic change (41). Secondly, pre-existing relationships thwarted the creation of a safe, non-judgemental space, restricting the disclosures that were made, and inhibiting group members to offer objective support and advice. Jung (53) compared the group therapeutic space to an alchemical “sealed container”, in which the group processes must be fully enclosed in order for therapeutic change to occur. There needs to be a clear and boundaried separation between what happens inside the group and what happens outside of it (52). Pre-existing relationships permeate these boundaries, bringing the outside world into the group, and generating the potential for discussions which take place within the confines of the group to impact on the outside world.

Finally, a culture of openness, support and empowerment was necessary to encourage sharing. Similar to findings by Buston et al. (8), if one mother was open and reflective, this could create an enabling group culture and encourage personal disclosures from the wider group. In contrast, mothers whose adverse experiences were raw or unprocessed could inhibit wider group sharing. Reflecting on childhood experiences can be painful, particularly for participants who have experienced abuse or neglect in childhood. A range of previous evaluations have confirmed that the effectiveness of group-based programmes is dependent on individual readiness for change (43–45), with some parents needing to undertake therapy to process their childhood experiences before being able to participate in a reflective group programme.

### 4.1 Limitations

There are a number of limitations to this research. First, a small sample of participants were interviewed from only three Mellow Babies groups, limiting the generalisability of findings. There is a huge variety of group and individual contextual factors which may interact and impact on mothers' experiences of Mellow Babies. A wider variety of experiences should be explored to provide a more comprehensive picture of how group context impacts programme delivery. Interviews were not conducted with two out of the three mothers who withdrew from the programme, and further research is needed to explore the experiences of participants who choose not to continue with group-based programmes.

A further limitation is that interviews elicit the subjective views of respondents and may not always represent “*truthful expression*” (54) pp. 30. Participant perspectives on their experiences are shaped by their self-knowledge, which even in the most self-aware individuals is incomplete and subject to blind spots (54). They may also be influenced by participant defence mechanisms. Hollway and Jefferson (55) describe interviewees as “*defended subjects*” whose subjective realities are shaped by *unconscious conflict, social discourses* and *psychic defences* (56). Participants may have found it difficult to acknowledge difficult experiences within the group, for example, within Group 2, one mother's difficulties due to her pre-existing relationship with another mother was acknowledged by all other group members interviewed, but not by the mother herself. Ivey (2023) expands this by describing the “*defended intersubjectivity*” to incorporate the unconscious biases which also influence researcher interpretation of participant discourses.

Finally, alongside the group context, the wider delivery context impacts programme efficacy, including practitioner skillset and the service delivery context. Practitioners within this study were relatively inexperienced, and this may have contributed to participants' experience of the group dynamics. Experienced practitioners are able to adopt a “dual attentiveness” (57) pp. 397, focusing on both delivering session content and facilitating supportive group dynamics. The wider service delivery context can also impact on the effectiveness of programmes, with the most successful programmes embedded within a wider support system, for parents and practitioners (16). The Mellow Babies delivery within this study was based on a temporary infrastructure set up solely for trial purposes. Findings may not be representative of real-world delivery. Also, as only these groups consisted solely of mothers, it is unknown whether findings will generalise to other group compositions. While certain group processes, for example the stages of group development, may apply universally, some may be specific to mothers, for example social identification from the shared challenges of motherhood.

### 4.2 Implications

Where possible, practitioners should give careful consideration to the composition of their group to ensure an optimum group size, sufficient balance of homogeneity and heterogeneity of background and experience, and to minimise the risk of pre-existing relationships inhibiting group dynamics. During the referral process, conversations are necessary with potential group members to ascertain their background and communicate clearly what the reflective discussions within the programme will involve. This will help ensure participants are psychologically prepared to benefit from the programme, and minimise the risk of adverse effects.

Other programme implementation implications could include a stronger focus on enabling group dynamics and managing group processes within programme manuals, training and supervision (57, 58). Employing a “spare” practitioner to cover for absences may prevent unplanned session cancellations. Findings also suggest that if there is going to be a week or two off (e.g., to coincide with the school term) this should be timetabled to occur later in the life of the group, to allow bonds to solidify and the group to reach the “performing” phase of group development. The ending of the group should be managed carefully, particularly when mothers have experienced a strong identification with the group, with a more gradual transition to group termination. It may be helpful to encourage groups to set up a WhatsApp chat, so they are able to continue bonding and offering support. However, further research is needed to explore whether group practitioners should be included or excluded from this.

### 4.3 Conclusions

Understanding how a programme may be affected by group context can offer valuable insight into the conditions under which the intervention is most effective, following the principles of realist evaluation (13). This study demonstrates that four group contextual factors impacted on the quality of Mellow Babies delivery across the three groups studied: (1) group size; (2) level of group homogeneity; (3) pre-existing relationships; (4) personalities within the group. Participants perceived that the most effective groups would:

• Have a size of between five and eight participants;
• Have a balance between homogeneity and heterogeneity of background and experiences;
• Have mothers who had no prior connections to other group members before the group;
• Be made up of group members who had sufficiently processed their own adversity so they were able to fully participate in reflective discussions and be supportive and empathic to others.

Group contextual factors affected the enabling group dynamics: (1) fluid progression through the stages of group development; (2) a safe, non-judgemental, contained space; (3) social identification with group; (4) group cohesion; and (5) a culture of openness, support and empowerment.

These findings have implications for the future delivery of Mellow Babies and other therapeutic group-based parenting programmes, highlighting the importance of consideration to group composition during programme recruitment. Practitioners may also benefit from a stronger focus on group processes and enabling group dynamics within training, supervision, and the programme manual. Future research is needed to explore a wider variety of experiences and contexts, and to explore how different contextual factors may interact.

## Data Availability

The raw data supporting the conclusions of this article will be made available by the authors, without undue reservation.
